# The Institute of Preventive Medicine

**Published:** 1893-02-25

**Authors:** Lawson Tait


### THE INSTITUTE OF PREVENTIVE MEDICINE.

Sir,—As you have been good enough to send me a marked copy of your issue of February 18th, in which the first lines are to the effect that I have expressed my entire approval of the objects of this Institute, I trust that you will permit me to say that this does not at all represent the attitude which I took, nor what I said. You very naturally say that if I approve of the objects can I avoid approving of the methods? That is the crux, and therefore I gave a reluctant and expectant assent to the resolution which was passed. Permit me to say that I am not an absolute and irreconcilable opponent of what is called Vivisection, but it is one of those things which requires the most complete justification for its employment. It has been so abused in the past, and has been so misleading, that I am exceedingly suspicious of any proposal for it in the present.—I am, &c.,

February 20th, 1893. Lawson Tait.

